# Experimental Hyperthyroidism Decreases Gene Expression and Serum Levels of Adipokines in Obesity

**DOI:** 10.1100/2012/780890

**Published:** 2012-05-03

**Authors:** Renata de Azevedo Melo Luvizotto, André Ferreira do Nascimento, Maria Teresa de Síbio, Regiane Marques Castro Olímpio, Sandro José Conde, Ana Paula Lima-Leopoldo, André Soares Leopoldo, Antonio Carlos Cicogna, Célia Regina Nogueira

**Affiliations:** ^1^Department of Internal Medicine, Botucatu School of Medicine, University of São Paulo State, 18618-000 Botucatu, SP, Brazil; ^2^Department of Sports, Center of Physical Education and Sports, Federal University of Espirito Santo (UFES), 29075-910 Vitória, ES, Brazil

## Abstract

*Aims*. To analyze the influence of hyperthyroidism on the gene expression and serum concentration of leptin, resistin, and adiponectin in obese animals. *Main Methods*. Male *Wistar* rats were randomly divided into two groups: control (C)—fed with commercial chow ad libitum—and obese (OB)—fed with a hypercaloric diet. After group characterization, the OB rats continued receiving a hypercaloric diet and were randomized into two groups: obese animals (OB) and obese with 25 **μ**g triiodothyronine (T_3_)/100 BW (OT). The T_3_ dose was administered every day for the last 2 weeks of the study. After 30 weeks the animals were euthanized. Samples of blood and adipose tissue were collected for biochemical and hormonal analyses as well as gene expression of leptin, resistin, and adiponectin. *Results*. T_3_ treatment was effective, increasing fT_3_ levels and decreasing fT_4_ and TSH serum concentration. Administration of T_3_ promotes weight loss, decreases all fat deposits, and diminishes serum levels of leptin, resistin, and adiponectin by reducing their gene expression. *Conclusions*. Our results suggest that T_3_ modulate serum and gene expression levels of leptin, resistin, and adiponectin in experimental model of obesity, providing new insights regarding the relationship between T_3_ and adipokines in obesity.

## 1. Introduction

The thyroid hormones influence energetic metabolism [1] and perform a central role in the regulation of adipose tissue metabolism [2]. Disturbances of these hormones are associated with alterations of body weight and energy expenditure [3]. While it is well known that hyperthyroidism leads to weight loss and hypothyroidism is associated with weight gain, the changes of thyroid function are discussed controversially in obesity [4].

Obesity, a public health problem associated with innumerable incapacitating and chronic diseases [5], is defined as an excessive or abnormal accumulation of adipose tissue that can be detrimental to health [6]. The adipose tissue, previously considered to be the largest, although inert, energy store in the body, actively produces a variety of biological substances. These substances are denominated adipokines and can influence the function and structural integrity of other tissues [7, 8]. In obesity, the release of adipokines such as leptin, resistin, and adiponectin can be altered [9]. Leptin acts as a signal of satiety in the hypothalamus and thus controls the body weight not only by diminishing the ingestion of foods but also by increasing energetic expenditure [10]. The levels of leptin in the circulatory system are elevated after meals; this increase is due to direct stimulation of expression of the *ob* gene and/or secretion of leptin from adipose tissue by glucose and insulin [11]. Resistin is secreted by monocytes and adipocytes, and present proinflammatory properties such as TNF-*α* and IL-6 [12]. Despite being expressed and secreted in thin individuals, elevated levels are associated with obesity both in humans and in experimental animal models [13]. However, the detailed functions of resistin are still not understood; it has been appreciated that resistin can cause hepatic insulin resistance and that it may, along with its closely related homologs, interact with immune cells as well [14]. Adiponectin is expressed exclusively in differentiated adipocytes [15], regulates lipid and glucose metabolism, suppresses gluconeogenesis, increases insulin sensitivity, stimulates fatty acid oxidation, protects against chronic inflammation, and regulates food intake and body weight [16]. Its reduction is associated with a decrease in lipid oxidation, increased triglycerides, and a suppression of insulin-dependent signaling in the liver and muscle, all of which can contribute to insulin resistance and obesity [17].

Thyroid hormones are involved in the regulation of adipose tissue whereas the hormones produced by adipose tissue such as resistin, adiponectin, and leptin are involved in regulation of the energetic balance [18]; however, the relationship between these hormones in obesity is controversial and scarcely addressed. In this context, our objective in this study was to analyze the influence of supraphysiological dose of T_3_ on the gene expression and serum concentration of leptin, resistin, and adiponectin in obese animals.

## 2. Materials and Methods

### 2.1. Animals and Experimental Protocol

This study utilized male *Wistar* rats, weighing approximately 150 g, supplied by the Animal Center of the Experimental Laboratory for Clinical Medicine at the “Júlio de Mesquita Filho” Paulista State University in Botucatu, Sao Paulo, Brazil. The animals were initially divided into two groups: control (C)—fed with commercial chow ad libitum—and obese (OB)—fed with a hypercaloric diet, as previously described [19], in order to induce obesity. After the obesity induction, the OB animals were randomized into two groups: obese animals (OB, *n* = 10), and obese animals administered a supraphysiological dose of T_3_ (OT, *n* = 10) at a concentration of 25 *μ*g/100 g body weight (BW) [20]. The T_3_ was administered by subcutaneous injections, once a day, during the final 2 weeks [21, 22]. Appropriate volumes of saline were administered, by subcutaneous injections, to the OB and C groups. The animals were housed in individual cages under controlled ambient temperature (22–26°C) and lighting (12 h light-dark cycle). Dietary consumption was controlled daily, and weight was assessed weekly. The experimental protocol was approved by the Commission for Ethics in Animal Experimentation at the Botucatu—UNESP School of Medicine, and followed the “Guidelines for the Care and Use of Experimental Animals.”

### 2.2. Total Body Fat

The total body fat was measured as the sum of epididymal, retroperitoneal, and visceral fat deposits [23]. This data point was utilized to confirm obesity in the animals. In addition, the adiposity index (total body fat divided by final body weight multiplied by 100, adapted from Boustany et al. [24]) was calculated.

### 2.3. Biochemical Analysis of Serum

The animals were fasted for 12 to 15 hours, anesthetized with sodium pentobarbital, 50 mg/kg/ip, and sacrificed by decapitation. The blood was collected in dry tubes and centrifuged at 3000 rpm for 10 minutes. The serum was stored at −80°C. Serum concentrations of glucose and triacylglycerol (TG) were assayed using specific kits (CELM, São Paulo, Brazil). Free fatty acids (FFAs) were determined using a commercial kit (WAKO, WAKO Pure Chemical Industries Ltd., Osaka, Japan). Dosing was analyzed by the automated colorimetric enzyme method (Technicon, RA-XT System, Global Medical Instrumentation, Minessota, USA).

### 2.4. Hormonal Measurements

Serum concentrations of insulin, leptin, resistin, adiponectin, free T_3_, free T_4_, and TSH were measured in all animals. The measurements were performed by immunoassay, measured with a microplate reader (Spectra Max 190—Molecular Devices, Sunnyvale, CA, USA). Commercial kits were utilized for the measurement of leptin, insulin, adiponectin (ELISA kit-Millipore, St. Charles, MO, USA), resistin (ELISA kit-B-Bridge International Inc., Mountain View, CA, USA), and thyroid hormones (ELISA kit-USCN Life Science&Technology Company, Wuhan, China).

### 2.5. Gene Expression

Whole RNA was extracted from retroperitoneal adipose tissue using the reagent Trizol (Invitrogen, Sao Paulo, Brazil), according to the manufacturer's instructions. The* SuperScript II First-Strand Synthesis System for RT-PCR* (Invitrogen, Sao Paulo, Brazil) kit was utilized for the synthesis of 20 *μ*L of complementary DNA (cDNA) from 1000 ng of whole RNA. The mRNA levels of leptin (assay Rn 00565158_mL—Applied Biosystems), resistin (assay Rn 00595224_mL—Applied Biosystems), and adiponectin (assay Rn 00595250_mL—Applied Biosystems) were determined by real-time PCR. Quantitative measurements were made with the commercial kit TaqMan qPCR (Applied Biosystems), according to the manufacturer's instructions, in the detection system Applied Biosystems StepOne Plus. Cycling conditions were as follows: enzyme activation at 50°C for 2 min, denaturation at 95°C for 10 min, the cDNA products were amplified for 40 cycles of denaturation at 95°C for 15 s, and annealing/extension at 60°C for 1 min. Gene expression was quantified in relation to the values of the C group after normalization by an internal control (cyclophilin-assay Rn 00690933_mL—Applied Biosystems) by the method 2^−ΔΔCT^, as previously described [25].

### 2.6. Statistical Analysis

Changes in body weight were evaluated by a confidence interval of 95%. Gene expression, biochemistry, and hormone data were analyzed using analysis of variance (ANOVA) complemented by Bonferroni's test. The data are expressed as mean ± standard deviation. A 5% significance level was adopted.

## 3. Results

### 3.1. Evolution of Body Weight

**Figure 1:**
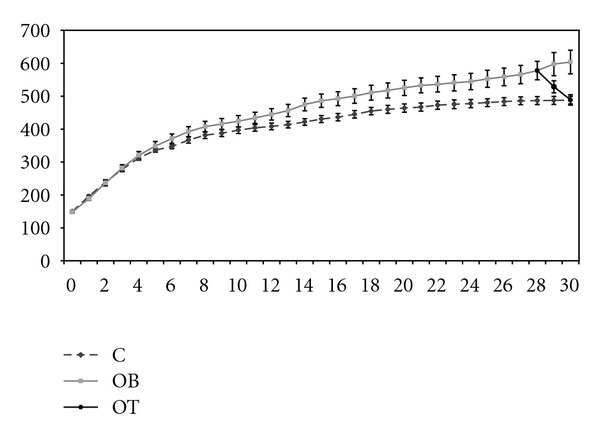
Weekly evolution of body weight in the control group (C, *n* = 10), obese group (OB, *n* = 10), and obese with 25 *μ*g T_3_/100 g BW (OT, *n* = 10). Data are expressed as means with a 95% confidence interval.

All the animals had similar BW at the beginning of the study. By week 13, the OB animals were heavier than the C group. After 30 weeks of experiment, the BW of the OB group (604 g ± 36 g) was statistically higher than that of the C group (488 g ± 11 g). The BW of the OB + T_3_ group (489 g ± 16 g) was significantly lower than the OB group, but not significantly different from the C group (Figure 1).

### 3.2. Total Body Fat

 The hypercaloric diet increased fat deposits and the adiposity index. Administration of T_3_ decreased retroperitoneal, visceral, and epididymal fat deposits. Similarly, T_3_ administration decreased total body fat and adiposity index (Table 1).

### 3.3. Biochemical Analysis

Table 2 presents the values for glucose, TG, and FFA. The hypercaloric diet did not alter the biochemical parameters when compared to the C group. There was an increase in glucose and FFA levels, but there was no statistical difference in TG levels in the OT group in comparison to the OB group.

### 3.4. Hormonal Measurements

**Figure 2:**
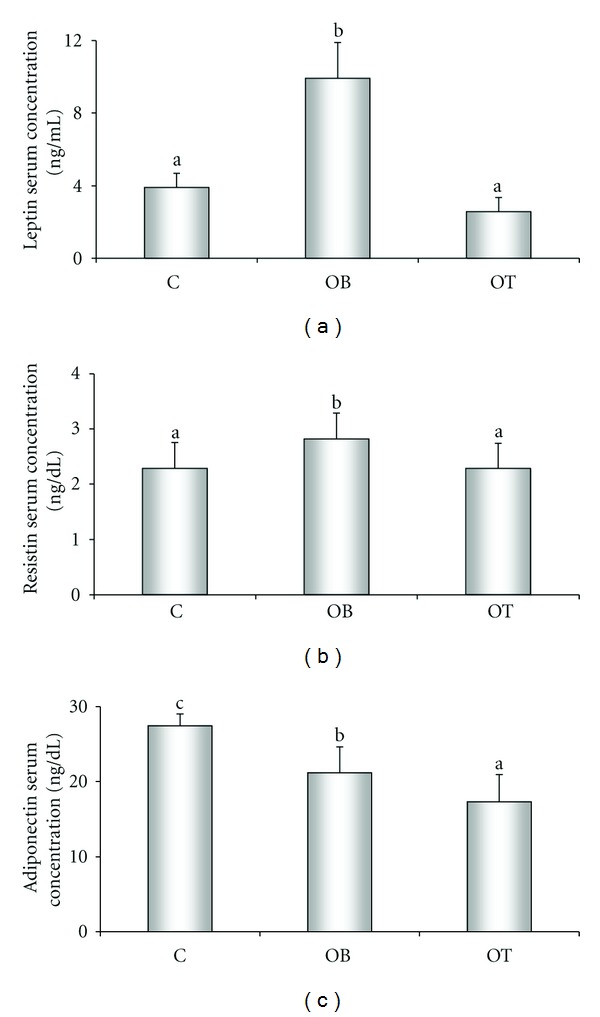
Influence of different doses of T_3_ on serum concentration of leptin (a), resistin (b), and adiponectin (c). C: control (*n* = 10); OB: obese (*n* = 10); OT: obese with 25 *μ*g T_3_/100 g BW (*n* = 10). Data expressed as mean ± standard deviation. ANOVA was utilized, complemented by Bonferroni's test. Use of same letters represent *P* > 0.05; different letters represent *P* < 0.05.

The hypercaloric diet did not change the thyroid profile; however, an increase in leptin and resistin levels and a decrease in adiponectin serum concentration was observed. In the OT group, T_3_ levels were elevated, yet the serum concentrations of free T_4_ and TSH were diminished (Table 3). Administration of T_3_ reduced the serum levels of adipokines, evaluated leptin (Figure 2(a)), resistin (Figure 2(b)), and adiponectin (Figure 2(c)).

### 3.5. Gene Expression

**Figure 3:**
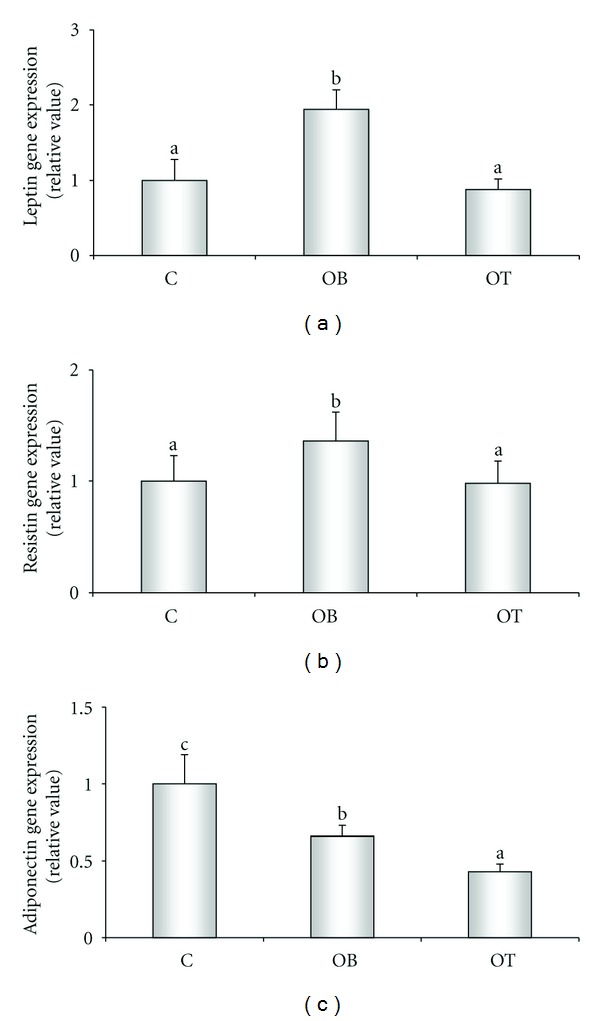
Influence of different T_3_ doses on the gene expression of leptin (a), resistin (b), and adiponectin (c). C: control (*n* = 6); OB: obese (*n* = 6); OT: obese with 25 *μ*g T_3_/100 g BW (*n* = 6). Data expressed as mean ± standard deviation. ANOVA was utilized, complemented by Bonferroni's test. Use of same letters represent *P* > 0.05; different letters represent *P* < 0.05.

Using real-time PCR, gene expression was analyzed using 6 animals per group. The samples were normalized by an internal control (cyclophilin), and the C group was normalized by 1. The gene expression of leptin (Figure 3(a)) and resistin (Figure 3(b)) was increased in the OB group, but diminished in the OT group. Compared to the C group, adiponectin gene expression (Figure 3(c)) was decreased by hypercaloric diet. Compared to the OB group, T_3_ administration decreased adiponectin expression levels.

## 4. Discussion

Obesity is a condition that has reached epidemic levels in recent years [6]. It is a complex disease, where lifestyle interacts with genetic susceptibility to produce the obese phenotype. Nowadays, the substantial rise in obesity indices appears to be due to the lifestyle of the population, particularly due to inappropriate diets and the lack of physical activity [4]. Lifestyle is highly recognized as playing a central role in the etiology of chronic diseases [26]. Furthermore, obesity is associated with several chronic diseases including coronary arterial disease, hypertension, diabetes mellitus type 2, and some forms of cancer [5].

The homogenous behavior of the animals is not assured in experimental studies, even when they are maintained under laboratory conditions. In this context, rats, given normocaloric or hypercaloric rations in models of diet-induced obesity, can present different responses with common characteristics [27]. Thus, classification errors may occur, such that animals submitted to a normocaloric diet can be classified as controls, when in fact they exhibit responses similar to animals that became obese via a hypercaloric diet, or vice versa. For this reason, it becomes necessary to establish a criterion that would enable the separation of animals into control or obese. A study in our laboratory showed that the best indicator of obesity is bodily adiposity, but this index is obtained after the animal is euthanized [27]. However, BW, evaluated in vivo, presents a good correlation with the adiposity index [27]. In this context, the control and obese groups were constituted by applying BW as the classification criterion of the study.

Administration of supraphysiological doses of T_3_ augmented the serum concentration of T_3_ [28]. In the OT animals, free T_4_ and TSH levels were diminished compared to OB animals, not surprisingly as exogenous T_3_ suppresses the endogenous secretion of TSH and T_4_ by the thyroid [29], showing the effectiveness of treatment.

Hypercaloric diets induce accentuated weight gain and adiposity. Consumption of diets rich in fat does not augment lipid oxidation in the same proportion, which leads to the elevation of body weight due to the deposition of triacylglycerol in adipose tissue [30, 31]. The supraphysiological doses of T_3_ diminished the weight of OB animals and reduced the adiposity index (Figure 1, Table 1). Hormones and cytokines induce distinct metabolic responses in different fat deposits [32], and this study shows a similar mobilization of all fat deposits in the OT group (Table 1).

Thyroid hormones regulate the metabolism of lipids [33] and that their excess, among other effects, augments lipolysis, the plasmatic concentration of intermediate lipids and lipid peroxidation [34]. Concentrations of free fatty acids are used to indicate the mobilization of fat [35]. In the present study, T_3_ administration elevates lipolysis in the OT group but did not alter TG levels (Table 2). These data are in agreement with other reports of TG levels not being influenced by thyroid hormone [36].

Excess of thyroid hormones augments plasma glucose levels [37]. The administration of T_3_ produced a significant diminution of plasma insulin levels in the animals treated (data not shown). Nevertheless, the exact influence of thyroid hormones on insulin sensitivity and glucose metabolism remains controversial [38]. However, one important factor to highlight in this study is that the thyroid hormones induce weight loss in a manner related to improving of resistance to insulin (data not shown) [39].

Leptin can influence the interaction between genes and environmental factors. Diets rich in fat raise leptin levels can differentially affect body composition even with similar diets. However, the rise in leptin levels is better explained by increase in body fat [31]. Experimental studies suggest that sensitivity to leptin can be controlled by hormonal and nutritional factors [40]. The literature shows a clear positive correlation between adipose tissue and leptin expression. Diet-induced obesity elevates the gene expression of leptin [41, 42]. However, the effects of T_3_ on the gene expression of leptin present inconsistent results; despite the in vitro data showing that T_3_ produces a dose-dependent rise in leptin expression [43], our data reveal that, following in vivo hyperthyroidism, leptin gene expression was reduced. In concordance, Pinkney et al. [2] and Zabrocka et al. [28] observed a diminution of leptin expression in response to treatment with T_3_.

The physiological role of adiponectin has not yet been completely elucidated. Experimental data suggest that adiponectin augments sensitivity to insulin and can present antiatherogenic and antiinflammatory properties [44]. It is well established that adiponectin levels are inversely proportional to the degree of adiposity [45], and weight loss elevates the endogenous production of adiponectin [46]. Here we demonstrate that the OB group had decreased adiponectin serum levels when compared to the C group, and the administration of T3, interestingly, even diminishing the body fat mass, presented lower levels of adiponectin (Figures 2(c) and 3(c)). Confirming this data Cabanelas et al. [47] show reduced adiponectin gene expression in inguinal explants of normal rats; also, we have demonstrate, recently that adiponectin levels are decreased in calorie-restricted obese rats [48]. However, in contrast, an experimental study of rats with hyperthyroidism showed an important rise in serum adiponectin [49]. Our data show that supraphysiological T_3_ doses alter adiponectin expression in obesity, suggesting that T_3_ causes undesirable effects on adipose tissue.

Resistin prejudices glucose homeostasis and insulin action in mice [50, 51]. Thus, resistin may perform an intermediary role between obesity and insulin resistance in rodents although this role is still questioned in humans [52]. In this study, a hypercaloric diet increases the serum levels of resistin, while T_3_ treatment decreases it. The significant diminution of serum resistin in the OT group corroborates the first study performed on humans, in which patients with hyperthyroidism exhibited low serum resistin concentrations [53]. However, these initial findings contrast with subsequent studies that report high resistin levels in hyperthyroidism patients [54, 55], showing a divergence in the data. Nevertheless, resistin gene expression is almost undetectable in rats with hyperthyroidism [56]. Our results show that administering supraphysiological doses of T_3_ decrease resistin expression to the C group levels (Figure 3(b)).

## 5. Conclusion

The exogenous treatment with T_3_ is effective in augmenting serum levels of free T_3_ and diminishing concentrations of free T_4_ and TSH. Administration of T_3_ promotes weight loss and decreases adiposity. Following administration of T_3_, there was a decrease in serum concentration of leptin, resistin, and adiponectin, as well as a reduction in the gene expression. Our data demonstrate that T_3_ acts, directly or indirectly, on adipose tissue-derived adipokines which can influence whole-body homeostasis. This report provides new insights regarding the relationship between T_3_ and adipokines in obesity model.

## Figures and Tables

**Table 1:** Composition of body fat: retroperitoneal, epididymal, visceral, and total fat deposits, and adiposity index.

Variable		Groups	
	C	OB	OT
Epid. fat (g)	8.65 ± 1.8^a^	13.3 ± 2.6^b^	7.0 ± 1.5^a^
Retro fat (g)	9.68 ± 3.2^a^	22.1 ± 6.0^b^	9.1 ± 3.9^a^
Visc. fat (g)	6.37 ± 1.5^a^	12.9 ± 4.0^b^	5.9 ± 1.8^a^
Total fat (g)	24.7 ± 5.6^a^	47.7 ± 10.8^b^	22.0 ± 6.8^a^
Adipos. I.	5.06 ± 1.1^a^	7.6 ± 1.0^b^	4.2 ± 1.4^a^

Epid. Fat: epididymal fat; Retro. Fat: retroperitoneal fat; Visc. Fat: visceral fat; total Fat: total body fat; Adipos. I: adiposity index; C: control; OB: obese; OT: obese with 25 *μ*g T_3_/100 g BW. Data expressed as mean ± standard deviation. ANOVA was utilized, complemented by Bonferroni's test. Use of same letters represent *P* > 0.05; different letters represent *P* < 0.05.

**Table 2:** Biochemical analysis: glucose, triglycerides, and free fatty acids.

Variable	Groups		
	C	OB	OT
Glucose (mg/dL)	95.0 ± 5.2^a^	93.2 ± 6.4^a^	103.1 ± 9.7^b^
TG (mg/dL)	82.5 ± 17.8^a^	82.2 ± 15.9^a^	73.2 ± 14.8^a^
FFA (mmol/L)	0.51 ± 0.1^a^	0.54 ± 0.1^a^	0.72 ± 0.1^b^

TG: triglycerides; FFA: free fatty acids; C: control; OB: obese; OT: obese with 25 *μ*g T_3_/100 g BW. Data expressed as mean ± standard deviation. ANOVA was utilized, complemented by Bonferroni's test. Use of same letters represent *P* > 0.05; different letters represent *P* < 0.05.

**Table 3:** Hormonal measurement of free triiodothyronine (T_3_), free thyroxine (T_4_), and thyroid-stimulating hormone (TSH).

Variable	Groups		
	C	OB	OT
Free T_3_ (pmol/L)	0.13 ± 0.06^a^	0.12 ± 0.03^a^	0.22 ± 0.04^b^
Free T_4_ (pmol/L)	45.8 ± 1.2^b^	44.8 ± 1.2^b^	37.8 ± 1.8^a^
TSH (mIU/L)	13.9 ± 0.9^b^	13.3 ± 0.8^b^	11.0 ± 1.2^a^

TSH: thyroid stimulating hormone; T_3_ triiodothyronine; T_4_ thyroxine; C: control; OB: obese; OT: obese with 25 *μ*g T_3_/100 g BW. Data expressed as mean ± standard deviation. ANOVA was utilized, complemented by Bonferroni's test. Use of same letters represent *P* > 0.05; different letters represent *P* < 0.05.
